# COVID-19 vaccine uptake and vaccine hesitancy in rural-to-urban migrant workers at the first round of COVID-19 vaccination in China

**DOI:** 10.1186/s12889-023-15068-1

**Published:** 2023-01-20

**Authors:** Liuzhi Hong, Zhou Jin, Kewei Xu, Guanghui Shen, Yang Zou, Ran Li, Lu Xu, Dexuan Wang, Li Chen, Yili Wu, Weihong Song

**Affiliations:** 1 School of Mental Health, Wenzhou Medical University, Wenzhou, China; 2 Institute of Aging, Key Laboratory of Alzheimer’s Disease of Zhejiang Province, Zhejiang Provincial Clinical Research Center for Mental Disorders, School of Mental Health and The Affiliated Kangning Hospital, Wenzhou Medical University, Oujiang Laboratory (Zhejiang Lab for Regenerative Medicine, Vision and Brain Health), Wenzhou, 325000 Zhejiang China; 3 The Second Affiliated Hospital and Yuying Children’s Hospital, Wenzhou Medical University, Wenzhou, China

**Keywords:** COVID-19 vaccine, Vaccine uptake, Vaccine hesitancy, Rural-to-urban migrant workers, VHDM

## Abstract

**Background:**

Migration can be linked to the transmission of COVID-19. COVID-19 vaccine uptake and hesitancy among rural-to-urban migrant workers in China, the largest group of internal migrants in the world, has not been characterized.

**Objective:**

To investigate COVID-19 vaccine uptake and identify vaccine hesitancy-associated factors among rural-to-urban migrant workers in the first round of COVID-19 vaccination in China.

**Methods:**

A cross-sectional questionnaire-based survey was conducted, including 14,917 participants. Socio-demographics, COVID-19 vaccine uptake, vaccine hesitancy and its associated factors based on Vaccine Hesitancy Determinants Matrix (VHDM) were applied for the survey. Data were principally analyzed by logistic regression analysis.

**Results:**

The COVID-19 vaccine uptake and vaccine hesitancy rates were 7.1% and 57.7%, respectively. Vaccine hesitancy was strongly associated with VHDM, including individual factors (female, higher annual income and fewer medical knowledge), group factors (less family support, friend support and public opinion support), COVID-19 epidemic factors (lower fatality, infection and emotional distress) and vaccine factors (less vaccine necessity, vaccine safety, vaccine efficacy, vaccine importance and vaccine reliability).

**Conclusion:**

The VHDM model has the potential utility in efforts to reduce COVID-19 vaccine hesitancy. Greater efforts should be put into addressing positive predictors associated with vaccine hesitancy.

## Background

The coronavirus disease 2019 (COVID-19) is the largest and most challenging pandemic in the twenty-first century, causing a devastating threat to global health and economy [1, 2]. To date, more than 213 countries have come under the attack of the COVID-19 pandemic and more than 51 million individuals have been infected including at least 6.3 million deaths globally (WHO, 2022). In order to prevent the spread of COVID-19, Chinese governments have imposed various movement restrictions and lockdowns, including isolation, quarantine, school closures, travel restrictions, and cancellation of mass gatherings [3]. These interventions showed the effectiveness of controlling the COVID-19 pandemic, which might seriously affect social and economic development. In addition to movement restrictions and lockdown, vaccination that obtains herd immunity is a more fundamental way to control the epidemic. Therefore, the high coverage of COVID-19 vaccination is much crucial for controlling the COVID-19 pandemic [4–6].

However, vaccination hesitancy is particularly evident in the initial period of the COVID-19 vaccine for marketing [7]. Vaccination hesitancy is a “delay” in acceptance or refusal of vaccination despite the availability of vaccine service (WHO, 2014), which is regarded as the top ten health concern by WHO [8]. The number of studies on vaccine hesitancy has increased rapidly since 2020 and most studies mainly focus on the nature of vaccination hesitancy [9], disparities [10, 11], as well as challenges and solutions on vaccine hesitancy [12, 13] among different high-risk groups including healthcare workers [9, 14, 15], pregnant women [16] and college students [17]. It is important to note that a range of determinants or predictors of vaccine hesitancy has been identified from individual differences [18], social environmental factors [19], and vaccine factors [20]. These studies highly indicate that vaccination hesitancy is a critical barrier to achieving the recommended COVID-19 vaccine coverage in non-mobile population. However, how is the prevalence and predictors of vaccination hesitancy in mobile population remains unclear.

First, we have chosen Chinese rural-to-urban migrant workers as our subjects. Rural-to-urban migrant workers, who migrate from rural areas of their original residence to urban areas for work-seeking, are a unique mobile group appearing in developing countries when experiencing economic transformation [21]. According to the National Bureau of Statistics of China, there were 290 million rural-to-urban migrant workers in 2020, making up more than one-third of the entire workforce in China [22]. Since the beginning of 2020, the end of the COVID-19 lockdown in China, massive migrant workers have poured into cities from place to place with constant changes in job or living conditions. The high mobility of migrant workers makes it difficult to monitor COVID-19 vaccination among this group and even leads to an increased risk of COVID-19 transmission if COVID-19 carriers exist. Moreover, COVID-19 vaccination hesitancy was associated with lower education levels, lower income, and health literacy [23], which are the most remarkable characteristics of migrant workers in China. However, a study on migrants in Shanghai, China reported a high acceptance of COVID-19 vaccination, no matter their socio-demographic characteristics [24]. Whether the cultural or regional differences contribute to such discrepancy in the association between vaccination hesitancy and individual factors remains unclear. Therefore, migrant workers may be a high-risk but neglected group of increased hesitancy for COVID-19 vaccination that deserves to be further investigated.

Second, we have chosen the first round of COVID-19 vaccination as the time of the investigation. The first COVID-19 vaccination campaign (referred to, in this paper, as the “first round”) was conducted from December 2020 to March 2021 when the COVID-19 vaccine was first approved for marketing in a few countries such as the UK, U.S, and China. It is a time not only under the urgent situation of COVID-19 prevention and control but under the uncertain situation of COVID-19 vaccine safety and effectiveness. As of December 13, 2022, the latest data showed that the COVID-19 vaccination coverage was 92.7% in China, and 90.4% had been fully vaccinated [25]. In first round of COVID-19 vaccination, however, a national survey showed that only 67.1% reported willingness and 35.5% were hesitant to accept vaccination [26]. The vaccine coverage was 34.4% in January 2021, which is far from the requirements of herd immunity [26]. Therefore, exploring the COVID-19 vaccination hesitancy of rural-to-urban migrant workers at this time is quite necessary and meaningful, which can generate more timely intervention on vaccination promotion for future epidemic events.

Finally, we would further attempt to understand and categorize the risk and protective factors of vaccination hesitancy based on the WHO’s Vaccine Hesitancy Determinants Matrix (VHDM) conceptual frameworks. VHDM model grouped the factors of vaccine hesitancy into four categories: individual factors, group factors, epidemic factors, and vaccine factors. Specifically, individual factors include personal characteristics (i.e., medical knowledge, preventive measures); the group factors include peer characteristics (i.e., friends support, family support); epidemic factors include epidemic characteristics (i.e., fatality, infection); vaccine factors include vaccine characteristics (i.e., vaccine necessity, safety, efficacy, importance, and vaccine reliability). For example, personal characteristics, including male, being married and healthy lifestyle were found to be associated with vaccine acceptance in China [27, 28]. Another study from China found that vaccine factors (i.e. trust in vaccine safety, effectiveness, access and price) appears to play a role in vaccine hesitancy [29]. To date, much of the existing literature on vaccine hesitance focuses on one of the above categories, whereas there is a lack of systematization and wholeness in research on it. VHDM model was drawn on adaptations of ecological models of health behavior to identify the multiple and interrelated levels of influence impacting vaccine hesitancy (Sturm 2005; Callréus 2010; WHO 2013; Larson 2014). Thus, it is theoretically and practically important to explore the factors of vaccine hesitancy based on the VHDM model to offer specific recommendations to translate COVID-19 vaccine hesitancy into acceptance and uptake.

To address the above research gaps, we: (1) investigate the coverage of COVID-19 vaccination in a large sample of 14,917 rural-to-urban migrant workers; (2) explore vaccination hesitancy in rural-to-urban migrant workers in the first round of COVID-19 vaccination; and (3) identify the determinants of vaccination hesitancy among migrant workers based on VHDM model.

## Methods

### Participants

We aimed to recruit a population of rural-to-urban migrant workers from China in the first round of COVID-19 vaccination. The inclusion criteria were: age ≥ 18 years, and possess an “agricultural” hukou status but work as non-agricultural workers in the urban areas [30]. Participants with unclear or missing responses to the variables of interest were excluded from the study. Finally, a total of 14,917 valid samples were collected.

### Procedure

A cross-sectional online survey was conducted among rural-to-urban migrant workers using a structured questionnaire in the first round of COVID-19 vaccination (from January 2 to March 2, 2021). The final eligible participants were identified by the following steps.

Firstly, in the pilot study, the pre-test was conducted with a convenience sample of 30 rural-to-urban migrant workers from the target population to evaluate the clarity, comprehensiveness, and acceptability of the questionnaires. Some amendments were made before the initial delivery. Secondly, All the participants were recruited and noticed by the local committee of economic and information technology who provides coverage of all the employees in all the companies in Zhejiang Province. Thirdly, the multistage probability sampling method was used. In stage 1, Wenzhou city of Zhejiang Province was chosen since Wenzhou was one of the most economically developed cities in China’s four economical zones and one of the most affected cities in terms of the number of COVID-19 cases apart from those in the hardest-hit Hubei Province. In stage 2, sixteen companies in each of the three districts were randomly selected. In stage 3, the cluster sampling method was used in each company. In total, 15,021 out of 16,000 respondents agreed and completed the online questionnaire by WeChat (the most popular social media platform in China). Among them, 104 were excluded due to a quality issue or logical error. Finally, 14,917 questionnaires underwent data analysis, with a valid response rate of 99.0%.

The questionnaires were anonymous and all participants took part in the study voluntarily. This study was conducted in compliance with the Helsinki Declaration and was reviewed and approved by the Ethics Committee of Wenzhou Medical University (Research Ethics Approval Code: 2021-K-23–02).

### Measures

The structured questionnaire contained three parts of information: (1) factors associated with COVID-19 vaccine hesitancy based on the VHDM model including individual and group factors, COVID-19 epidemic factors, and vaccine factors; (2) outcome variables including the status of COVID-19 vaccination and vaccination hesitancy; (3) quality control questions.

#### Factors associated with COVID-19 vaccine hesitancy

##### Individual factors

A questionnaire elicited individual basic background information including gender, age, education level, marital status, average annual household income, and medical knowledge.

##### Group factors

Group factors include the vaccinated environment (someone around has been vaccinated) and socio-support environments, such as family support, friend support, unit support, government support, and public opinion support.

##### COVID-19 epidemic factors

COVID-19 epidemic characteristics factors include (1) risk perception of COVID-19, such as fatality, worry to be infected (worry much that oneself or a family member would contract the disease); (2) the negative effect of COVID-19; (3) emotional distress (Feeling much in panic or much depressed or much emotionally disturb). These factors were designed to assess the contextual influence on COVID-19 vaccination hesitancy.

##### Vaccine factors

Vaccine characteristics factors were defined as specific issues directly related to COVID-19 vaccine, which assessed the vaccine-specific influence on vaccination hesitancy including the vaccine necessity, safety, efficacy, importance to families or himself/herself, importance to others, vaccine reliability, and vaccine recommendation. Each vaccine factor was assessed by one question. Specifically, vaccine necessity is measured by the item “It is very necessary for you to get the COVID-19 vaccine”, safety by the item “Although COVID-19 vaccine is a new type of vaccine, its safety is beyond doubt”, efficacy by the item “I think COVID-19 vaccine can effectively prevent COVID-19”, importance to families or himself/herself by the item “COVID-19 vaccines are very important for my health and my family’s healthy”, importance to others by the item “COVID-19 vaccines are very important for the health of others”, vaccine reliability by the item “COVID-19 vaccines offered in the local hospitals are beneficial”, and vaccine recommendation by the item “I do what my doctor recommends about getting COVID-19 vaccines’ recommendation.”

#### Outcome variables

##### COVID-19 vaccine uptake

The experience of a COVID-19 vaccine uptake was measured using a one-item question (Have received at least one COVID-19 vaccine dose?) with a “yes” or “no” response.

##### COVID-19 vaccine hesitancy

The COVID-19 vaccine hesitancy was measured using a one-item question (If a vaccine for COVID-19 was available for you, would you take it?) with “yes”, “not sure” or “no” as possible responses. If the participants answered “not sure”, they were considered a vaccine hesitancy group; If the participants answered “yes”, they were considered a vaccine acceptance group; If the participants answered “no”, they were considered a vaccine rejection group.

#### Quality control

We monitored the progress of the survey every day. After the deadline, we checked the accuracy of the data. Two quality control questions (“What is the current year?” and “When is China’s National Day?”) were set for detecting inattentive samples. Questionnaires would be excluded if logical contradictions were detected in the answers. In order to avoid the interference from “too fast” responses, we removed the individuals who completed the survey in a short period of time (less than 3 min, which we considered the minimum time needed to complete a valid survey).

### Statistical analysis

The socio-demographic characteristics of the sample were described by the number and the percentage of each category. Bivariate analyses including cross-tabulation analysis and independent samples t-test were conducted to evaluate the characteristics of COVID-19 vaccine uptake and hesitancy among rural-to-urban migrant workers. Then, a multiple logistic regression model with the maximum-likelihood estimation of parameter values was used to determine the independent contribution of the VHDM model to COVID-19 vaccine hesitancy. We incorporated 24 relevant covariates (including individual factors, group factors, COVID-19 epidemic factors and vaccine factors) into our analysis as independent variables, with COVID-19 vaccine hesitancy as the dependent variable. Adjusted odds ratios and 95% confidence intervals were derived from logistic-regression coefficients to provide an estimate of the statistical association between a given variable and acceptance of COVID-19 vaccine(s) when the other variables held constant. All statistical analyses were performed by using the SPSS statistics package (version 25.0) and all reported *P*-values are 2-tailed with a statistical significance of 0.05.

## Results

### Socio-demographic characteristics of participants

Data collected showed that the age of the participants ranged from 18 to 75 years old (*M* = 35.50, *SD* = 8.93 years) with a majority aged 30 to 50 years. Of these more than half (58.2%) were males while 42.8% were females. Almost half (42.1%) of participants had an education level of junior high school or below and most (71.2%) were married. The annual income of 89.3% of participants was 50 thousand RMB (approximately US$7470) or below. The general demographic characteristics of all participants are summarized in Table 1.

**Table 1:** Characteristics of COVID-19 vaccination among rural-to-urban migrant workers

	All participants (*n* = 14,917)	COVID-19 Vaccination		*p*
		Yes (*n* = 1066)	No (*n* = 13,851)	
**Individual factors**				
Gender				0.01
Male	8680 (58.2%)	658 (7.6%)	8022 (92.4%)	
Female	6237 (41.8%)	408 (6.5%)	5829 (93.5%)	
Age (years)				< .001
25 or below	2026 (13.6%)	85 (4.2%)	1941 (95.8%)	
26–35	5917 (39.7%)	343 (5.8%)	5574 (94.2%)	
36–45	4672 (31.3%)	398 (8.5%)	4274 (91.5%)	
46 or above	2302 (15.4%)	240 (10.4%)	2062 (89.6%)	
Education level				< .001
Junior high school or below	6287 (42.1%)	545 (8.7%)	5742 (91.3%)	
High school /Secondary	3550 (23.8%)	238 (6.7%)	3312 (93.3%)	
College or above	5080 (34.1%)	283 (5.6%)	4797 (94.4%)	
Marital status				< .001
Single	3757 (25.2%)	184 (4.9%)	3573 (95.1%)	
Married	10,624 (71.2%)	824 (7.8%)	9800 (92.2%)	
Others	536 (3.6%)	58 (10.8%)	478 (89.2%)	
Annual household income				0.06
Below 30 thousand	2013 (13.5%)	168 (8.3%)	1845 (91.7%)	
30–50 thousand	5513 (37.0%)	400 (7.3%)	5113 (92.7%)	
50–100 thousand	5791 (38.8%)	381 (6.6%)	5410 (93.4%)	
Above 100 thousand	1600 (10.7%)	117 (7.3%)	1483 (92.7%)	
Medical knowledge				< .001
Yes	1117 (7.5%)	143 (12.8%)	974 (87.2%)	
No	13,800 (92.5%)	923 (6.7%)	12,877 (93.3%)	
Past vaccinated by choice experience				< .001
Yes	5885 (39.5%)	568 (9.7%)	5317 (90.3%)	
No	9032 (60.5%)	498 (5.5%)	8534 (94.5%)	
**Group factors**				
Someone around has been vaccinated				< .001
Yes	4313 (28.9%)	549 (12.7%)	3764 (87.3%)	
No	10,604 (71.1%)	517 (4.9%)	10,087 (95.1%)	
Family support				< .001
Yes	11,117 (74.5%)	944 (8.5%)	10,173 (91.5%)	
No	3800 (25.5%)	122 (3.2%)	3678 (96.8%)	
Friend support				< .001
Yes	11,192 (75.0%)	945 (8.4%)	10,247 (91.6%)	
No	3725 (25.0%)	121 (3.2%)	3604 (96.8%)	
Unit support				< .001
Yes	12,537 (84.0%)	976 (7.8%)	11,561 (92.2%)	
No	2380 (16.0%)	90 (3.8%)	2290 (96.2%)	
Government support				< .001
Yes	12,912 (86.6%)	990 (7.7%)	11,922 (92.3%)	
No	2005 (13.4%)	76 (3.8%)	1929 (96.2%)	
Public opinion support				< .001
Yes	11,923 (80.0%)	952 (8.0%)	10,976 (92.0%)	
No	2989 (20.0%)	114 (3.8%)	2875 (96.2%)	
**COVID-19 epidemic factors**				
High fatality of COVID-19				0.001
Disagree	3000 (20.1%)	252 (8.4%)	2748 (91.6%)	
Unsure	3737 (25.1%)	286 (7.7%)	3451 (92.3%)	
Agree	8180 (54.8%)	528 (6.5%)	7652 (93.5%)	
Negatively affected by COVID-19				0.001
Barely	2040 (13.7%)	140 (6.9%)	1900 (93.1%)	
General	4259 (28.6%)	255 (6.0%)	4004 (94.0%)	
Largely	8618 (57.8%)	671 (7.8%)	7947 (92.2%)	
Worry much that oneself or family member would contract the disease				0.38
Yes	12,595 (84.4%)	910 (7.2%)	11,685 (92.8%)	
No	2322 (15.6%)	156 (6.7%)	2166 (93.3%)	
Emotional distress (Feeling much in panic or much depressed or much emotionally disturbed)				< .001
Yes	4781 (32.1%)	398 (8.3%)	4383 (91.7%)	
No	10,136 (67.9%)	688 (6.6%)	9468 (93.4%)	
**Vaccine factors**				
Vaccine necessity	3.75 ± 1.10	3.83 ± 1.24	3.75 ± 1.09	0.03
Vaccine safety	3.89 ± 0.89	4.12 ± 0.89	3.87 ± 0.88	< .001
Vaccine efficacy	4.00 ± 0.82	4.19 ± 0.83	3.99 ± 0.81	< .001
Vaccine importance for families or myself	4.15 ± 0.83	4.37 ± 0.80	4.13 ± 0.83	< .001
Vaccine importance for others	4.15 ± 0.81	4.33 ± 0.82	4.14 ± 0.81	< .001
Vaccine Reliability	3.98 ± 0.83	4.24 ± 0.82	3.96 ± 0.83	< .001
Vaccine recommendation	4.27 ± 0.80	4.36 ± 0.81	4.27 ± 0.80	< .001

### Prevalence and characteristics of COVID-19 vaccine uptake

COVID-19 vaccine uptake and characteristics of vaccinated and unvaccinated respondents were shown in Table 1. Of 14,917 participants, only 1066 (7.1%) subjects have been vaccinated against COVID-19 and 13,851 (92.9%) have not got the COVID-19 vaccination.

From the individual perspective, COVID-19 vaccine uptake showed significant associations (*p* < 0.01) with males, older age (46 years or above), a lower education level (junior high school or below), married, more medical knowledge, and past vaccinated by choice experience. From the group perspective, COVID-19 vaccine uptake showed the strongest associations (*ps* < 0.001) with the detection of close people already vaccinated and a supportive environment from family, friends, unit, government, and public opinion. From the perspective of COVID-19 epidemic characteristics, COVID-19 vaccine uptake was significantly correlated (*ps* < 0.001) with the COVID-19 epidemic characteristics, including perceived high fatality, largely affected by COVID-19, and emotional distress of COVID-19. From the perspective of COVID-19 vaccine characteristics, COVID-19 vaccine uptake was significantly associated with perceived higher vaccine necessity, safety, efficacy, importance, reliability, and recommendation (*ps* < 0.05). (Detail information is displayed in Table 1).

### Prevalence and characteristics of COVID-19 vaccine hesitancy

Among 13,851 participants who were not vaccinated, 4853 (35.0%) reported intention of vaccine uptake, 396 (2.9%) reported refusal of COVID-19 vaccines and 8602 (62.1%) reported “not sure” about their intention.

From the individual perspective, COVID-19 vaccine hesitancy showed the strongest associations (*ps* < 0.001) with a female, younger age, higher education level, and less medical knowledge. From the group perspective level, COVID-19 vaccination hesitancy was significantly correlated (*ps* < 0.001) with lower supportive environments ( e.g. family support, friend support, unit support, government support, and public opinion support). From the perspective of COVID-19 epidemic characteristics, COVID-19 vaccination hesitancy was significantly correlated (*p* < 0.001) with risk perception and emotional distress during the COVID-19 epidemic. Specifically, a significantly higher proportion of respondents who perceived high fatality (40.6%) expressed vaccine acceptance (*p* < 0.001). Respondents who suffer emotional distress such as feeling much in panic or much depressed (43.7%) or worry about infection (38.1%) declared a strong acceptance of COVID-19 vaccination when compared to those without feeling panic (32.5%) or less worry about infection (24.8%). From the perspective of COVID-19 vaccine characteristics, subjects who perceived lower vaccine necessity, safety, efficacy, importance, reliability, and recommendation expressed more hesitancy toward COVID-19 vaccination (*p* < 0.001). Details of vaccination hesitancy were displayed in Table 2.

**Table 2:** Characteristics of COVID-19 vaccination hesitancy among rural-to-urban migrant workers

	Vaccination acceptance (*n* = 4853)	Vaccination hesitancy (*n* = 8602)	*p*	aOR (95%CI)	*p*
**Individual factors**					
Gender			< .001		
Male	3001 (38.6%)	4765 (61.4%)		1 (ref)	
Female	1852 (32.6%)	3837 (67.4%)		**1.24 (1.11–1.39)**	** < .001**
Age (years)			0.003		
25 or below	611 (32.4%)	1274 (67.6%)		1 (ref)	
26–35	1961 (36.3%)	3448 (63.7%)		0.92 (0.76–1.12)	0.41
36–45	1527 (36.7%)	2629 (63.3%)		1.05 (0.85–1.31)	0.65
46 or above	754 (37.6%)	1251 (62.4%)		1.15 (0.91–1.46)	0.25
Education level			< .001		
Junior high school or below	2227 (39.6%)	3393 (60.4%)		1 (ref)	
High school /Secondary	1287 (39.8%)	1949 (60.2%)		0.99 (0.86–1.13)	0.85
College or above	1339 (29.1%)	3260 (70.9%)		1.06 (0.91–1.24)	0.43
Marital status			< .001		
Single	1116 (32.4%)	2324 (67.6%)		1 (ref)	
Married	3562 (37.3%)	5987 (62.7%)		0.91 (0.78–1.07)	0.27
Others	175 (37.6%)	291 (62.4%)		0.92 (0.67–1.26)	0.59
Annual household income			< .001		
Below 30 thousand	681 (38.3%)	1095 (61.7%)		1 (ref)	
30–50 thousand	1867 (37.5%)	3118 (62.5%)		1.15 (0.97–1.35)	0.11
50–100 thousand	1884 (35.8%)	3384 (64.2%)		**1.22 (1.03–1.46)**	**0.02**
Above 100 thousand	421 (29.5%)	1005 (70.5%)		**1.41 (1.09–1.81)**	**0.008**
Medical knowledge			< .001		
Yes	390 (41.6%)	547 (58.4%)		1 (ref)	
No	4463 (35.7%)	8055 (64.3%)		**1.26 (1.04–1.54)**	**0.02**
Past vaccinated by choice experience			< .001		
Yes	2099 (40.4%)	3102 (59.6%)		1 (ref)	
No	2754 (33.4%)	5500 (66.6%)		1.10 (0.98–1.22)	0.10
**Group factors**					
Someone around has been vaccinated			0.65		
Yes	1308 (35.8%)	2350 (64.2%)		1 (ref)	
No	3545 (36.2%)	6252 (63.8%)		0.96 (0.85–1.08)	0.46
Family support			< .001		
Yes	4258 (42.4%)	5795 (57.6%)		1 (ref)	
No	595 (17.5%)	2807 (82.5%)		**1.28 (0.99–1.64)**	**0.05**
Friend support			< .001		
Yes	4276 (42.3%)	5839(57.7%)		1 (ref)	
No	577 (17.3%)	2763 (82.7%)		**1.39 (1.06–1.83)**	**0.02**
Unit support			< .001		
Yes	4437 (39.2%)	6896 (60.8%)		1 (ref)	
No	416 (19.6%)	1706 (80.4%)		0.81 (0.61–1.07)	0.13
Government support			< .001		
Yes	4471 (38.3%)	7190 (61.7%)		1 (ref)	
No	382 (21.3%)	1412 (78.7%)		0.81 (0.61–1.07)	0.14
Public opinion support			< .001		
Yes	4373 (40.6%)	6404 (59.4%)		1 (ref)	
No	480 (17.9%)	2198 (82.1%)		**1.28 (0.99–1.64)**	**0.05**
**COVID-19 epidemic factors**					
High fatality of COVID-19			< .001		
Disagree	860 (32.7%)	1772 (67.3%)		1 (ref)	
Unsure	951 (28.5%)	2382 (71.5%)		1.05 (0.90–1.24)	0.52
Agree	3042 (40.6%)	4448 (59.4%)		**0.82 (0.71–0.95)**	**0.007**
Negatively affected by COVID-19			< .001		
Barely	521 (28.8%)	1290 (71.2%)		1 (ref)	
General	1046 (26.9%)	2841 (73.1%)		0.95 (0.79–1.13)	0.55
Largely	3286 (42.4%)	4471 (57.6%)		**0.77 (0.65–0.91)**	**0.002**
Worry much that oneself or family member would contract the disease			< .001		
No	506 (24.8%)	1537 (75.2%)		1 (ref)	
Yes	4347 (38.1%)	7065 (61.9%)		**0.82 (0.70–0.97)**	**0.02**
Emotional distress			< .001		
Yes	1864 (43.7%)	2406 (56.3%)		1 (ref)	
No	2989 (32.5%)	6196 (67.5%)		**1.51 (1.34–1.70)**	** < .001**
**Vaccine factors**					
Vaccine necessity	4.23 ± 1.23	3.52 ± 0.88	< .001	**0.75 (0.71–0.78)**	** < .001**
Vaccine safety	4.54 ± 0.73	3.55 ± 0.69	< .001	**0.48 (0.44–0.52)**	** < .001**
Vaccine efficacy	4.60 ± 0.63	3.68 ± 0.66	< .001	**0.65 (0.59–0.72)**	** < .001**
Vaccine importance for families or myself	4.81 ± 0.46	3.80 ± 0.70	< .001	**0.32 (0.28–0.36)**	** < .001**
Vaccine importance for others	4.78 ± 0.50	3.82 ± 0.69	< .001	**0.48 (0.43–0.54)**	** < .001**
Vaccine Reliability	4.54 ± 0.72	3.68 ± 0.68	< .001	**0.87 (0.80–0.95)**	**0.001**
Vaccine recommendation	4.68 ± 0.68	4.06 ± 0.75	< .001	**0.73 (0.67–0.78)**	** < .001**

### The VHDM models of COVID-19 vaccine hesitancy

**Fig. 1:**
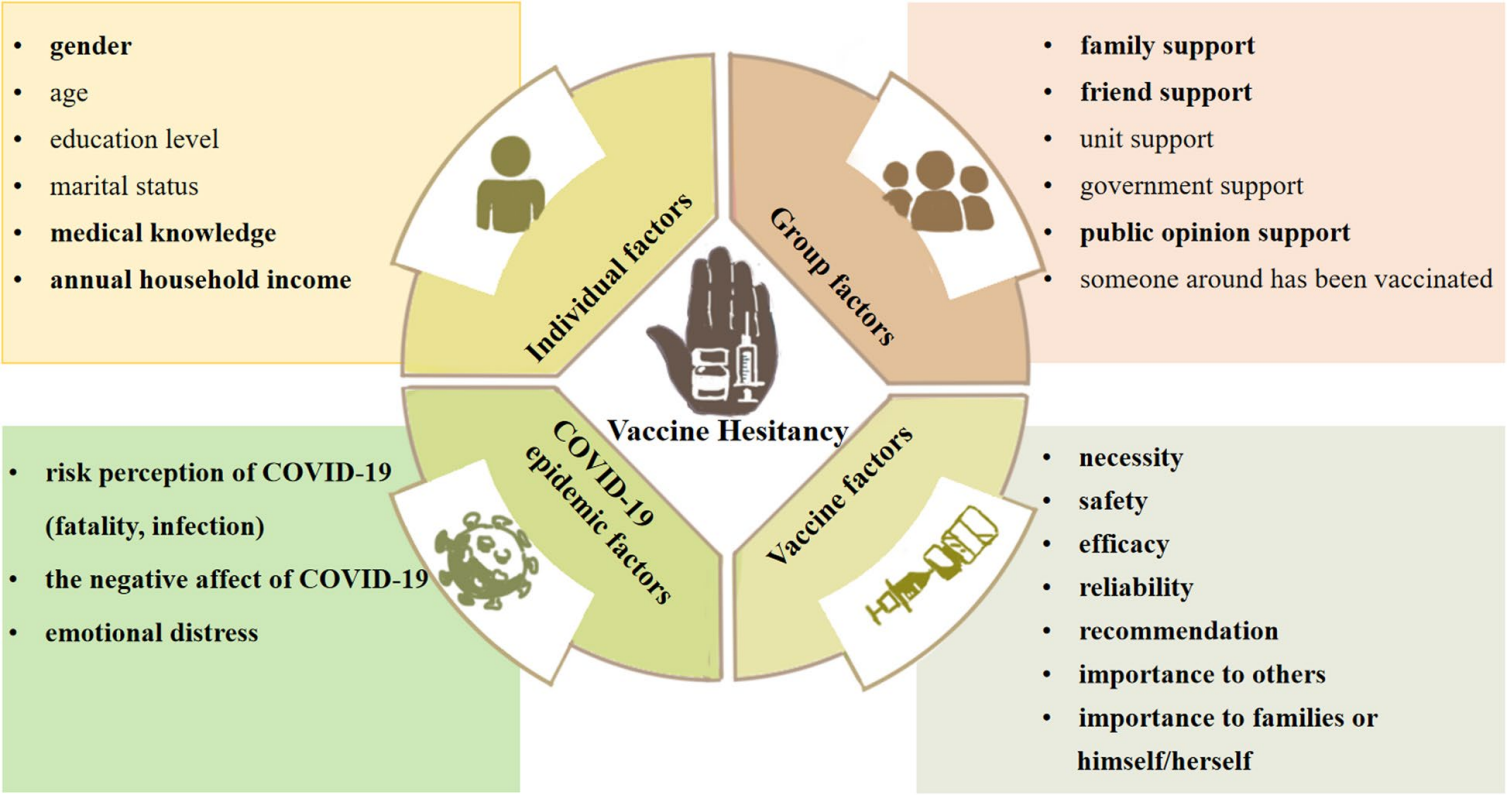
The Vaccine Hesitancy Determinants Matrix (VHDM) Model of Vaccine Hesitancy. The VHDM model defined four categories of factors associated with vaccine hesitancy, including individual factors, group factors, vaccine factors, and COVID-19 epidemic factors. Among these categories, the factors with statistical significance (*p* < 0.05) in the current study were shown in bold

According to the VHDM model, potential factors associated with vaccine hesitancy were analyzed. Moreover, the associated factors were identified by the multivariate logistic regression from four perspectives: individual factors, group factors, COVID-19 epidemic factors, and vaccine factors (see Table 2 and Fig. 1).

From the individual perspective, the logistic regression analysis for vaccine hesitancy revealed that females (OR: 1.24; 95% CI: 1.11–1.39) and respondents with a higher annual income (above 100 thousand) (OR: 1.41; 95% CI: 1.09–1.81) were more likely to respond “not sure” versus “yes” compared with male and respondents with lower annual income. Moreover, respondents who have less medical knowledge were 1.26 times more likely to respond “not sure” versus “yes” (95% CI: 1.04–1.54) (see Table 2).

From the group perspective, supportive environments including family support, friend support, and public opinion support, had significant negative associations with vaccine hesitancy. Specifically, respondents who perceived less friend support were more likely to respond “not sure” versus “yes” (OR: 1.39; 95% CI: 1.06–1.83). Similarly, subjects who perceived less family support and public opinion support were more likely to respond “not sure” versus “yes” (family support: OR: 1.28, 95% CI: 0.99–1.64; public opinion support: OR:1.29, 95% CI: 0.99–1.64) than those who perceived more family support, but with marginal significance (*p* = 0.05). Other group characteristics, such as unit support and government support did not consistently achieve statistical significance.

From the perspective of COVID-19 epidemic characteristics: respondents who perceived high fatality (OR: 0.82; 95% CI: 0.71–0.95), and high infection (OR: 0.82; 95% CI: 0.71–0.95) were significantly associated with responding “yes” for vaccination intentions (*p* < 0.05). Moreover, respondents who suffered emotional distress, such as feeling much in panic or much depressed or much emotional disturbance, or much worry because of the COVID-19 epidemic, were more likely to accept the vaccine. Specifically, subjects with less emotional distress were 1.51 times more likely to respond “not sure” versus “yes” (95% CI: 1.34–1.70).

From the perspective of COVID-19 vaccine characteristics, vaccine necessity, vaccine safety, vaccine efficacy, vaccine importance, vaccine reliability, and vaccine recommendation were strongly negatively associated with vaccine hesitancy. Respondents who perceived higher vaccine necessity (OR: 0.75; 95% CI: 0.71–0.78) and vaccine efficacy (OR: 0.65; 95% CI: 0.59–0.72) were more likely to accept COVID-19 vaccines. Moreover, vaccine safety and vaccine importance had a larger effect on vaccine hesitancy (ORs < 0.50) than on vaccine reliability and vaccine recommendation (ORs > 0.70). Specifically, respondents who perceived more vaccine importance for his/her families or himself/herself were 3.13 times more likely to accept the vaccine than those who perceived less vaccine importance (OR: 0.32; 95% CI: 0.28–0.36).

Overall, in the VHDM model of vaccine hesitancy, individual factors (ΔR^2^ = 0.03), group factors (ΔR^2^ = 0.05), contextual factors (ΔR^2^ = 0.03) and vaccine factors (ΔR^2^ = 0.35) explained 3%, 5%, 3% and 35% of variance hesitancy, respectively (see Fig. 1).

## Discussion

This is the first study to explore COVID-19 vaccine hesitancy among the largest mobile population in the world at the first round of COVID-19 vaccination. In this study, the prevalence and characteristics of COVID-19 vaccine uptake and vaccine hesitancy were estimated, then vaccine hesitancy-associated factors were identified at four levels based on the model of VHDM.

The first startling finding was that COVID-19 vaccine uptake among rural-to-urban migrant workers at the first round of COVID-19 vaccination was extremely low and only 7.1%, which was much lower than the estimated coverage required to achieve herd immunity (70.0% or above) and even the national level in China during the same period (34.4%—42%) [26, 31]. This finding suggested that the implementation of the COVID-19 immunization program had been inefficient among rural-to-urban migrant workers. Therefore, more specific and robust policies and regulations are needed to enhance COVID-19 immunization in urban areas where the migrant workers mostly flowinto. Importantly, COVID-19 vaccine uptake among rural-to-urban migrant workers was positively associated with middle age, marriage, low education level, more medical knowledge, and past vaccination by choice experience. These factors have often been reported in previous studies [18, 20]. Therefore, more attention should be paid to young or unmarried adults with highly educated, medical knowledge or without past vaccination experience for COVID-19 vaccination.

The second finding highlights the alarmingly high rate of COVID-19 vaccine hesitancy in rural-to-urban migrant workers after the vaccine was made available was reported up to 62.1%. After COVID-19 vaccines are on the market, there was a surge of interest in estimating the rate of COVID-19 vaccine hesitancy around the world [32]. A Systematic Review [33] including 31 studies on COVID-19 vaccine hesitancy in 33 different countries showed that the lowest COVID-19 vaccine hesitancy rates were found in Ecuador (3.0%), Malaysia (5.7%), Indonesia (6.7%) and China (9.7%). However, the highest COVID-19 vaccine hesitancy rates were found in Kuwait (76.4%), Jordan (71.6%), Italy (46.3), Russia (45.1%), Poland (43.7%), the US (43.1%), and France (41.1%). Our findings were unique in revealing the COVID-19 vaccine hesitancy in the population of migrant workers and its relatively higher rate compared with the world average. However, it remains unknown whether the high hesitancy of the COVID-19 vaccine among migrant workers in China results from geographical differences or this particular sub-population due to the lack of comparable data. Therefore, it is necessary to obtain more data for further validation in future studies.

The third important finding of the study is that four levels of determinants were significantly associated with COVID-19 vaccine hesitancy based on the model of VHDM including individual, group, COVID-19 pandemic, and vaccine factors.

From the individual perspective of the VHDM model, gender, annual income, and medical knowledge at the first round of COVID-19 vaccination were three of the strongest factors associated with COVID-19 vaccine hesitancy among rural-to-urban migrant workers. Specifically, our findings were in concordance with the literature [34], where females showed more unwillingness to accept the COVID-19 vaccine than males. A previous systematic review on the global influenza pandemic in 2009 also demonstrated that females were less likely to be vaccinated than males [35]. The reason for this may be that men engage in riskier behaviors than women [36] and women tend to collect medical information from various sources when it comes to their families' health [36]. Furthermore, women’s hesitancy to accept the COVID-19 vaccine may make vaccinating children difficult, as women play a key role in child vaccination when the COVID-19 vaccine is accessible to children [37]. Secondly, income level was also associated with vaccine hesitancy where those with higher income migrant workers were more likely to be vaccine hesitancy. In this regard, the available literature also cannot provide a consistent result [38]. This discrepancy could be related to various standard incomes used, different samples selected, and different data analyses performed in these studies. Finally, medical knowledge was a strong protective factor of vaccine acceptance. Since vaccination could be considered one of the most important preventive measures to protect against COVID-19 infection, people with more medical knowledge and high awareness of prevention would likely be more willing to get vaccinated.

From the group perspective of the VHDM model, supports from family, friend and public opinion would be significantly helpful to reduce vaccine hesitancy. These findings were consistent with previous studies [39–41] and support the views of the social-ecological theory that personal, family and social factors have a synergistic effect on individual's mental states and behavior [42]. Furthermore, vaccine hesitancy was a dynamic and potentially reversible state compared to avoidance or refusal and social support for vaccination is an important motivator for vaccine-hesitant individuals. As China is deeply influenced by collectivism, families, and friends have a profound influence on individuals’ emotions, behavior, and decision-making. Their guidance is a powerful component in the decision-making process. It should be noted that support from friends appears to be more important than from families, which is not consistent with the previous study on the general sample [39]. A possible explanation is that rural-to-urban migrant workers have to leave their families to work resulting in fewer connections with their families and weakening their families’ influence on their intention on COVID-19 vaccination. Thus, those with a low level of friend support were more likely to be vaccine hesitancy and vaccine refusal. Furthermore, respondents in the lower public opinion supportive environment are more likely to respond “no” versus “yes” to vaccine intention. It is clear that support from public opinion could increase the likelihood of stable trust in vaccine safety and effectiveness. As such, our study confirmed that a supportive environment seems to have a significant effect on the hesitant respondents.

From the COVID-19 epidemic perspective of the VHDM model, individuals who perceived the risk of the COVID-19 pandemic including high fatality, high infection, and emotional distress had a stronger intention to have the COVID-19 vaccine. This finding is consistent with a number of studies identifying the association between perceived COVID-19 infection risk and vaccine uptake and acceptance [43, 44]. Furthermore, our findings also confirmed the risk as feeling theory [45, 46], which maintains that people’s reactions to danger vary depending on the specific characteristic of a hazard. Specifically, if risks are perceived as more dangerous when they are uncommon and unknown to science, people would react in a positive and proactive way and vice versa. Therefore, the COVID-19 epidemic is likely to induce a high-risk perception as it is a new disease, for which both science and people have little or no information and experience, with a catastrophic nature, thus evoking strong feelings, which leads to an increase in the vaccine acceptance [47]. Meanwhile, those results confirmed the role of risk perception on judgment and decision-making in health care for a disease associated with serious consequences, uncertain outcomes, and limited scientific knowledge, showing how the perceived risk drives the decision to immunize.

From the COVID-19 vaccine perspective of the VHDM model, there is a strong positive association between all vaccine-specific factors and the intention to be vaccinated against COVID-19. Among them, vaccine safety and vaccine importance were the strongest factors associated with vaccine acceptance. Our result indicated that higher safety, effectiveness, necessity, and importance of vaccines will be critical to achieving high vaccine uptake among target populations especially in the early phases after the vaccine is on the market. Thus, public health initiatives should focus on increasing trust in vaccine safety and emphasize the importance of vaccines for individuals and society.

Our findings also indicate three implications. First, it is crucial for government and health authorities to formulate effective and appropriate vaccine policies and plans based on occupation, age, life characteristics, and VHDM. For example, the mandatory and accessibility of vaccination should be strengthened for high-risk occupational groups. Moreover, healthcare providers should disseminate transparent and accurate information about vaccines’ safety and efficacy to gain the trust of the population, especially those with vaccine hesitancy or refusal. As medical knowledge, and vaccine information were associated with vaccine acceptance, it is critical to make full use of multiple media to enhance the comprehensibility of vaccine information and to publicize vaccine safety, effectiveness, and importance. Finally, from the perspective of individuals, family and friend support contributed significantly to vaccination intentions.

The current study presents several strengths and limitations. A major strength is that we investigated the coverage of COVID-19 vaccination in a large sample of 14,917 rural-to-urban migrant workers, who might be particularly at risk in the COVID-19 pandemic. Furthermore, we explored vaccination hesitancy in rural-to-urban migrant workers in the first round of COVID-19 vaccination, a critical period of vaccination full of uncertainty. The current study was conducted in Wenzhou city of Zhejiang province, which allows for regional comparisons of migrant workers' vaccine hesitancy. Compared to another survey conducted in Shanghai, China [24], the rate of COVID-19 vaccine hesitancy was quite high (62.1%) in the current study. Moreover, our study identified the determinants of vaccination hesitancy among migrant workers based on VHDM model. While much research focused on one category of determinants, our study comprehensively measured individual, group, epidemic, and vaccine factors, which provided a systematic understanding on the associated factors of vaccine hesitancy.

We should acknowledge some limitations in our study. First, this study was based on a cross-sectional design, which was not possible to get a valid cause-and-effect relation between COVID-19 vaccine hesitancy and the associated factors. Secondly, the questionnaires were published via WeChat and the data about vaccination uptake were collected by using participants’ own reports, instead of through healthcare facilities. This may result in information bias. We will make effort to collect data through more reliable facilities to confirm the reliability of vaccine uptake. Thirdly, most of the respondents were from Wenzhou, Zhejiang Province, which may lead to a selection bias. Finally, not all components in the model of VHDM were included, such as culture, political circumstance, race, etc.

## Conclusion

This study provides empirical evidence for the prevalence of COVID-19 vaccine intake and hesitancy in rural–urban migrant workers from the perspective of the VHDM model. Alarmingly, only 7.1% of migrant workers have been vaccinated against COVID-19, and up to 62.1% reported experiencing vaccine hesitancy. In conclusion, the uptake rate of the COVID-19 vaccine and vaccination intention was suboptimal to achieve herd immunity. It is urgent for governments, public health officials, and economic and social groups to develop a strategy to improve vaccination acceptance, especially in the working population. In this case, the VHDM model may serve as an effective tool for identifying determinants of vaccine hesitancy.

## Data Availability

All data used and/or analyzed in the present study are available from the corresponding author on reasonable request. They are not publicly available, in accordance with the Ethics Review Authority.

## Abbreviations

COVID-19: Coronavirus disease 2019
VHDM: Vaccine Hesitancy Determinants Matrix
